# Actual evapotranspiration by machine learning and remote sensing without the thermal spectrum

**DOI:** 10.1371/journal.pone.0285535

**Published:** 2023-05-11

**Authors:** Taiara Souza Costa, Roberto Filgueiras, Robson Argolo dos Santos, Fernando França da Cunha

**Affiliations:** Department of Agricultural Engineering, Center of Agricultural Sciences, Federal University of Viçosa, Viçosa-MG, Brazil; Universidade Federal de Uberlandia, BRAZIL

## Abstract

The objectives of this study were to use machine learning algorithms to establish a model for estimating the evapotranspiration fraction (ET_*f*_) using two data input scenarios from the spectral information of the Sentinel-2 constellation, and to analyze the temporal and spatial applicability of the models to estimate the actual evapotranspiration (ET_r_) in agricultural crops irrigated by center pivots. The spectral bands of Sentinel 2A and 2B satellite and vegetation indices formed the first scenario. The second scenario was formed by performing the normalized ratio procedure between bands (NRPB) and joining the variables applied in the first scenario. The models were generated to predict the ET_*f*_ using six regression algorithms and then compared with ET_*f*_ calculated by the *Simple Algorithm For Evapotranspiration Retrieving* (SAFER) algorithm, was considered as the standard. The results possible to select the best model, which in both scenarios was Cubist. Subsequently, ET_*f*_ was estimated only for the center pivots present in the study area and the classification of land use and cover was accessed through the *MapBiomas* product. Land use was necessary to enable the calculation of ET_r_ in each scenario, in the center pivots with sugarcane and soybean crops. ET_r_ was estimated using two ET_o_ approaches (ET_o_Brazil and Hargreaves-Samani). It was found that the Hargreaves-Samani equation overestimated ET_r_ with higher errors mainly for center pivots with sugarcane, where systematic error (MBE) ranged from 0.89 to 2.02 mm d^-1^. The ET_o_Brazil product, on the other hand, presented statistical errors with MBE values ranging from 0.00 to 1.26 mm d^-1^ for both agricultural crops. Based on the results obtained, it is observed that the ET_r_ can be monitored spatially and temporally without the use of the thermal band, which causes the estimation of this parameter to be performed with greater temporal frequency.

## 1. Introduction

With the advancement of technologies and with the aid of agrometeorological models aimed at the determination of evapotranspiration (ET), it becomes possible to estimate, spatially and accurately, the water demand of agricultural crops [1]. Such information is essential in irrigated agriculture. Thus, ET is a key parameter in the water balance and, in this context, the use of data from remote sensing stands out [2–4]. These estimates are generally based on the components of the energy balance equation and have shown a high degree of reliability and speed in ET estimation [5].

Among the agrometeorological models, it is possible to mention the Simple Algorithm For Evapotranspiration Retrieving (SAFER) presented by Teixeira [6]. SAFER is an energy balance algorithm developed based on the modeling of evapotranspiration fraction (ET_*f*_), of easy application and operability, calibrated in the field [7, 8], and has good results in studies with emphasis on irrigation management [4]. For Roerink et al. [9], ET_*f*_ is the ratio between the energy released in the form of latent heat to the sum of latent heat and sensible heat.

To estimate ET_*f*_ through the SAFER algorithm, images from the Landsat-8 satellite have been used [6]. This is possible because, the Landsat-8 satellite has an electromagnetic spectrum of the thermal infrared region. This electromagnetic spectrum makes it possible to estimate the surface temperature, being considered one of the main parameters directly responsible for determining actual evapotranspiration (ET_r_) [10–12]. However, the temporal resolution of Landsat-8 (16 days) becomes an obstacle when the goal is to carry out irrigation management.

Given the above, the need to study other satellites with better temporal resolution is clear. However, there are no free satellites available that combine high spatial and temporal resolution, in addition to the ability to capture electromagnetic radiation emitted in the thermal infrared region, which could make irrigation management accessible by satellite images. On the other hand, Sentinel 2A and Sentinel 2B satellites have good spatial and temporal resolution, although they do not have the ability to capture electromagnetic radiation emitted in the thermal infrared region [13].

Considering this scenario, it is clear that there is a significant challenge for ET modeling with frequent images without the thermal infrared spectrum, which would help in the decisions related to management of water resources at the agricultural property level [14]. Therefore, it is desirable to develop techniques that make it possible to fill this gap by providing detailed and frequent information on ET_r_.

As an alternative and promising tool, there are machine learning algorithms that have increasingly been applied in ET modeling [15, 16]. These tools, in general, surpass the conventional equations for estimating ET_r_ and/or reference evapotranspiration (ET_o_) [17–19] and provide information that allows us to visualize how ET behaves in space and time [20, 21].

These machine learning algorithms are robust mathematical models with high predictive potential [22], and in addition, are able to capture complex relationships between data input and output, with interesting accuracy in the predicted values [3, 19]. It is noteworthy that machine learning algorithms are sensitive to input covariates. Therefore, it is important to use models capable of predicting ET with high performance, among them: *Multiple Linear Regression* (MLR), *Linear Support Vector Machine* (Linear SVM), *Cubist*, *Bayesian-Regularized Neural Network* (BRNN) and *eXtreme Gradient Boosting* (Xgb) by the *Linear* and *Tree* methods. More information on these methods can be found in the subtopic “Regression algorithms used in predicting ET_*f*_” in the material and methods item of this paper.

Although many studies have used algorithms to estimate ET, only Santos et al. [23] evaluated the impact of using machine learning algorithms in estimating ET_*f*_ using only surface reflectance data. These authors used information from the OLI and TIR sensors (Landsat 8) to calculate the ET_*f*_ in areas cultivated with sugarcane using the METRIC algorithm. The spectral bands of the MSI sensor, from the Sentinel-2 satellites, gave rise to the independent variables for input in the machine learning algorithms. With this, they concluded that algorithms were able to identify patterns in the MSI sensor data to predict the ET_*f*_ when comparing with the standard model, with the *XgbLinear* model showing the best results. The study by Santos et al. [23] demonstrates that machine learning equations have the potential to predict ET in a simpler way, as it does not need the principles of mass and energy estimated by fully physical models and also do not need the energy balance calculated by the thermal band. For El-Hendawy et al. [24], spectral information has been used with great success to detect even the small biophysical and biochemical variations and modifications of the plant canopy.

Based on this need for further studies and on the availability of the Sentinel-2 constellation, this study aimed to estimate ET_*f*_ through regression algorithms and spectral information from Sentinel 2A and Sentinel 2B satellites and to analyze the temporal and spatial applicability of ET_*f*_ obtained to estimate the ET_r_ in agricultural crops irrigated by center pivots.

## 2. Material and methods

### 2.1. Study area

**Fig 1:**
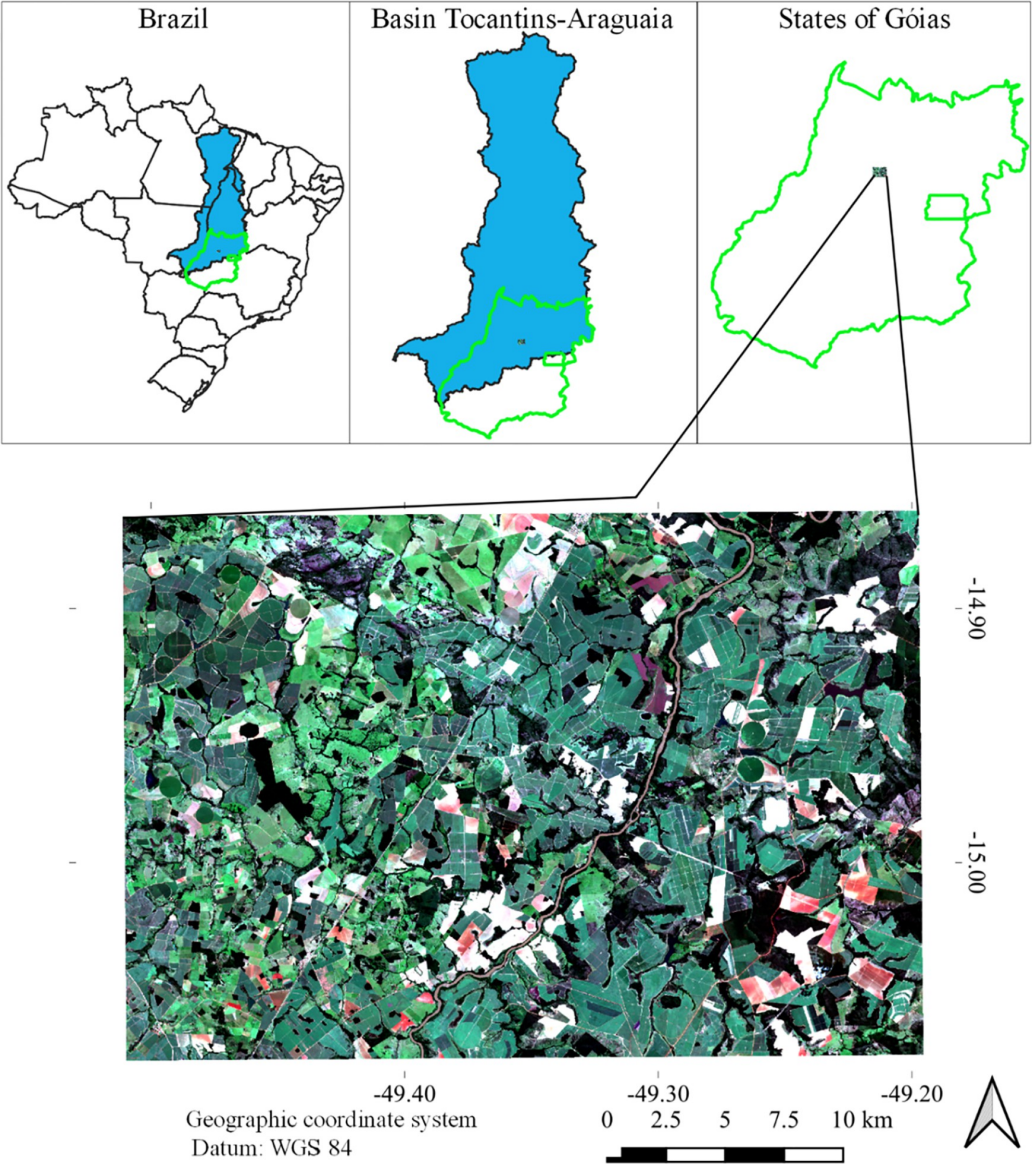
Geographic location referring to the area used to model the evapotranspiration fraction (ET_*f*_). The figure was elaborated using the open source software QGIS [25]—using an RGB image composite generated from the Landsat 8 satellite images that were downloaded from https://earthexplorer.usgs.gov/. *Landsat-8 image courtesy of the U*.*S*. *Geological Survey* according to [26].

The study was carried out in an area belonging to the hydrographic region of the Tocantins-Araguaia river (Fig 1), being located in the municipality of Itapaci, state of Goiás, delimited by the pairs of coordinates X1: 660185.23; Y1: 8356189.30 (upper left corner) and X2: 693932.13; Y2: 8332533.58 (lower right corner), DATUM WGS84 reference system—UTM plane coordinate system—Zone 22S.

The study area, including exposed soil, has the following land uses: native forest, pasture, rivers, irrigated agriculture and rainfed. The area under study was selected due to the high density of center pivots. Therefore, to ensure water security, irrigation management is necessary. On the other hand, irrigation management also contributes to food security, since water is fundamental to obtain agricultural products in greater quantity and quality [27, 28].

**Fig 2:**
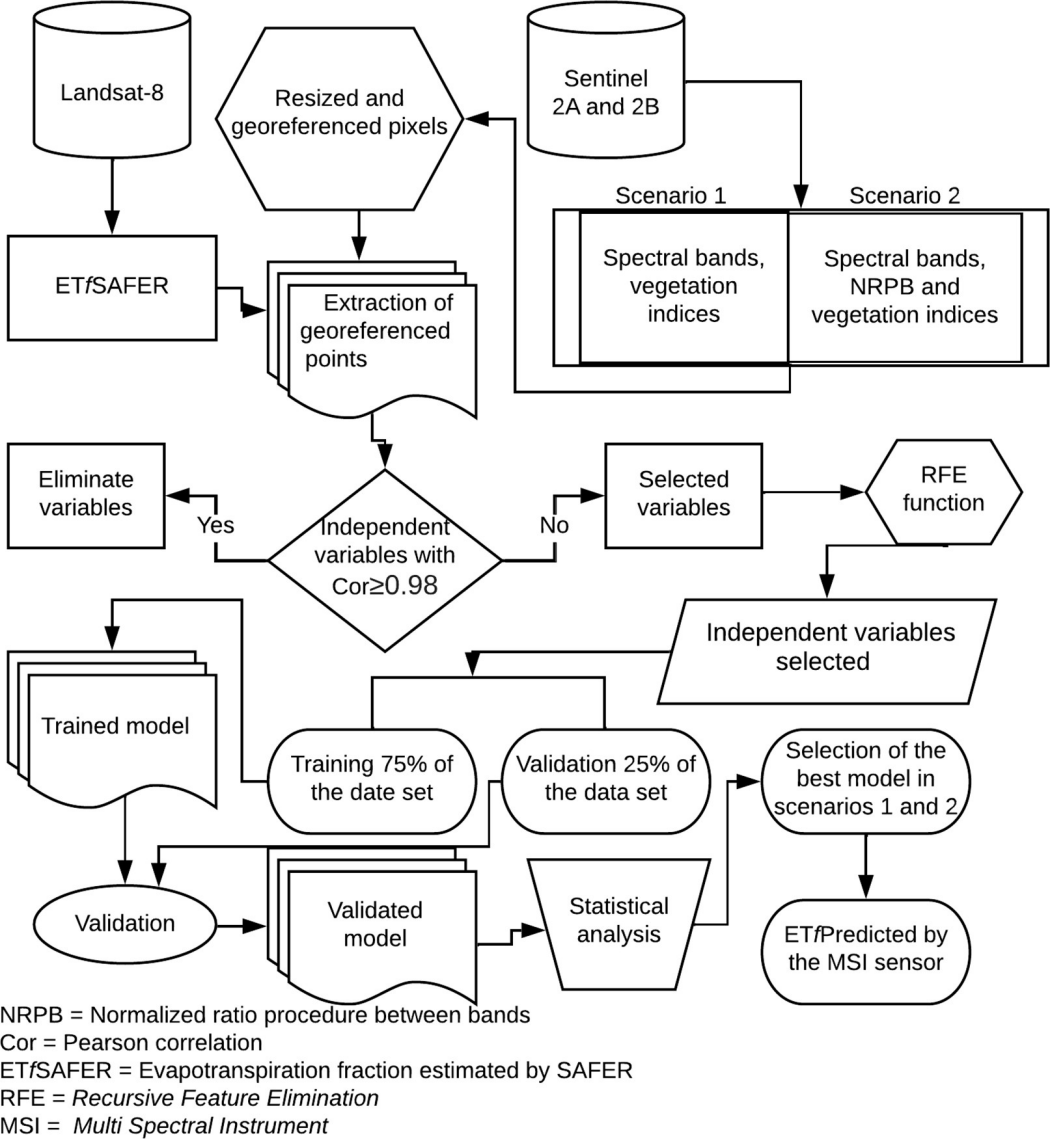
Methodology used to predict the evapotranspiration fraction (ET_*f*_).

Fig 2 shows the flowchart with the steps taken for the modeling of the target variable, which are later explained in the topics.

### 2.2. Regression algorithms used in predicting ET_*f*_

In an attempt to model ET_*f*_, six regression algorithms were used: *Multiple Linear Regression* (MLR), *Linear Support Vector Machine* (Linear SVM), *Cubist*, *Bayesian-Regularized Neural Network* (BRNN) and *eXtreme Gradient Boosting* (Xgb) by the *Linear* and *Tree* methods. All algorithms were implemented using the *Caret* package [29], in R *software* [30].

These models have a multivariate regression approach, in addition to a strong statistical basis, and have received increasing research interest in recent years [31]. However, the performance of each model may vary according to the characteristic of the variable to be modeled, which justifies the importance of its choices [31]. Regression algorithms are mathematical models that seek to find an equation that quantifies the relationship between a dependent variable (Y) and one or more independent variables (X_*1*_,…, X_*p*_). That is, its analysis is a prediction of the values of one or more response variables to which a set of explanatory variables is used [32, 33].

The regression algorithms used in the study will be described in a simplified way. However, more information about the models used can be obtained in [31]. The *Multiple Linear Regression* (MLR) equation with *p* explanatory variables is represented by Eq 1.


$$Y_{i}=B_{0}+B_{1xi1}+B_{2xi2}+\ldots +B_{\mathrm{pxip}}+\varepsilon_{i},\;i=1,\ldots ,n$$ (1)

Where *y*_*i*_ represents the value of the response variable in the observation *i*, *i* = 1,…,*n*; *xi*1, *xi*2,…*xip*, *i* = 1,…,*n* are the values of the *i*-ésima observation of the *p* explanatory variables; *B*_0_, *B*_1_, *B*_2_,…,*B*_*p*_ are the regression parameters or coefficients; *ε*_*i*_, *i* = 1,…,*n* correspond to random errors.

The *Linear Support Vector Machine* (Linear SVM) is obtained through Eq 2 and seeks to maximize the generalization ability and minimize structural risk. In addition, this model is not sensitive to outliers, that is, extreme values do not cause noise in the training of the data. The basic functioning of the SVM consists of adjusting the equation of a line, called a hyperplane, in such a way that the distance between it and points with different characteristics is maximized.


$$\vec{w}.\vec{x}‐b=\left\{ \begin{array}{c} ‐1,\;first\;support\;vector\\ 0,\;hyperplane\\ 1,\;second\;support\;vector \end{array}\right.$$ (2)

Where $\vec{w}$ is a vector perpendicular to the points; $\vec{x}$ is the vector of the set of points; b is an optional constant that can be used as a BIAS. When the result of this equation is equal to 1 or -1, it is one of the support vectors, with the result greater than 0 and less than 1 or less than 0 and greater than -1, it is a margin space.

The *Cubist* builds the model based on a set of unordered rules, where each rule is formed from conditions associated with a linear expression. The rules generated by *Cubist* have the following structure:

$$<R>:\;\left\{ \begin{array}{c} {}[N_{\mathrm{cob}},\;V_{m},\;I,\;E_{\mathrm{est}}]\\ if\;complex\\ then\;<class>=C \end{array}\right.$$ (3)

Where R represents the number of rules; N_cob_ is the number of examples covered by the rule; V_m_ is the average (predicted) meta-attribute value of the covered examples; I = [V_min_, V_max_] is a range of predicted values (minimum and maximum value) for the attribute-meta class; E_est_ is the expected error estimate; complex are the rule conditions R and C is the linear model (mathematical function) for the target attribute.

*Bayesian-Regularized Neural Networks* (BRNN) are trained using supervised learning, with a training dataset of inputs and destinations D = {(*x*_*1*_, *y*_*1*_), (*x*_*2*_, *y*_*2*_), … (*x*_*N*_, *y*_*N*_)}. In the Bayesian structure, the weights of the network are considered random variables [34]. With the data set, the probability density function of a matrix w of network weights is:

$$f(w|D,\;\propto ,\;\beta ,\;M)=\frac{f(D|,\;w,\;\beta ,\;M)f(w|,\;\propto ,\;M)}{f(D|\propto ,\;\beta ,\;M)}$$ (4)

Where M is the particular neural network model used; *f*(*w*|,∝,*M*) is the prior density, which represents our knowledge of the weights before any data is collected; *f*(*D*|,*w*,*β*,*M*) is the likelihood function, which is the probability of the data occurring given the weights; *f*(*D*|∝,*β*,*M*) is a normalization factor, which guarantees that the total probability is 1.

Xgb is a new simple tree-based ensemble method that was developed by Chen et al. [35], which is an improved version of gradient boosting with higher computational efficiency and better ability to deal with overfitting problems [35]. Xgb is formed by a set of classification and regression trees K (CART) {*T*_*1*_ (*x*_*1*_, *y*_*1*_) … *T*_*K*_ (*x*_*i*_, *y*_*i*_) where *x*_*i*_ is the provided training set of descriptors associated with a molecule to predict the *y*_*i*_ class label. Given that a CART assigns an actual score to each sheet (outcome or target), the prediction scores for individual CARTs are summed to obtain the final score and evaluated using *K* additive functions as shown in Eq 5.


$$\hat{Y_{i}}=\sum_{k=1}^{K}f_{k}(X_{i}),\;f_{k}\in F$$ (5)

Where *f*_*k*_ represents an independent tree structure with leaf scores and F is the space of all CART.

### 2.3. Target variable: evapotranspiration fraction (ET_*f*_)

To model the evapotranspiration fraction (ET_*f*_) from regression models and readily available information, images of Sentinel 2A and 2B satellites were used as input variables in the models. However, the information from Landsat-8 was used as a reference for the calculation of ET_*f*_ by Simple Algorithm For Evapotranspiration Retrieving [6], here called ET_*f*_SAFER.

The satellites used in the research were the target of study due to the potential in terms of spatial and temporal resolution, in addition to the existence of the band referring to the thermal spectrum present only in Landsat-8. Satellite images were acquired on the *Earth Explorer* platform (http://earthexplorer.usgs.gov) belonging to the United States Geological Survey [36]. After the download, a visual analysis was carried out to select images free of clouds and noises with a view to not compromising the analysis of the models. After this analysis, six pairs of images were selected (Landsat-8 and Sentinel-2) with the same dates, and they were recorded on 02/03/2016, 03/09/2017, 08/16/2017 and 01/23/2018, 08/06/2019 and 04/02/2020.

Then, a pre-processing was performed in order to leave the images ready to be used in the estimation of ET_*f*_. In this step, operational procedures were performed in QGis® *software* [25]. The Semi-Automatic Classification Plugin [37], was used for the conversion of digital numbers to physical values (Landsat-8 and Sentinel images) and for atmospheric correction (Landsat-8 and Sentinel images). The Dark Object Subtraction (DOS1) atmospheric correction methodology [38], was adopted to contain the additive effects caused by the dispersion of particles present in the atmosphere. Then, the coordinate system was reprojected to UTM, zone 22 South.

### 2.4. Methodologies: Resources adopted to model the target variable

For training regression algorithms, the four first images (02/03/2016, 03/09/2017, 08/16/2017 and 01/23/2018) of the six selected from the Sentinel-2 (A or B) and Landsat-8 satellites were used. ET_*f*_SAFER was used as dependent variable, calculated with Landsat-8 information from the reflectances of the bands (ρ_2_, ρ_3_, ρ_4_, ρ_5_, ρ_6_ and ρ_7_) and radiance ρ_10_ required to estimate biophysical parameters presented in the methodology of Teixeira [6]. On the website (https://eos.com/blog/band-combinations-for-landsat-8/) it is possible to access detailed information about each spectral band of the Landsat 8 satellite.

The independent variables were the reflectances of the multispectral bands from the Sentinel-2 constellation (ρ_2_, ρ_3_, ρ_4_, ρ_8_, ρ_5_, ρ_6_, ρ_7_, ρ_8A_, ρ_11_ and ρ_12_) and three different vegetation indices. The indices used were NDVI—normalized difference vegetation index [39], EVI—enhanced vegetation index [40], and NDWI—normalized difference water index [41], which were calculated according to Eqs 6, 7 and 8, respectively. NDVI and EVI are associated with vegetation growth stage, while NDWI is a moisture index.


$$\mathrm{NDVI}=\frac{\rho_{8}–\rho_{4}}{\rho_{8}+\rho_{4}}$$ (6)

$$\mathrm{NDWI}=\frac{\rho_{8}–\rho_{12}}{\rho_{8}+\rho_{12}}$$ (7)

$$EVI=G\frac{\rho_{8}-\rho_{4}}{{(\rho }_{8}+{(C}_{1}\times \rho_{4})–{(C}_{2}\times \rho_{2})+L}$$ (8)

Where *ρ*_*8*_, *ρ*_*4*_, *ρ*_*12*_, *ρ*_*2*_ are the reflectances of the near infrared (band 8), red (band 4), short wave infrared (band 12) and blue (band 2) bands of the sensor *Multi Spectral Instrument* (MSI); *G* is the adjusted gain factor; *L* is the soil adjustment factor; and *C*_*1*_ and *C*_*2*_ are coefficients for aerosol correction. For the coefficients *G*, *C*_*1*_, *C*_*2*_ and *L*, the values of 2.5, 6.0, 7.5 and 1.0 were assumed, respectively [40].

Sentinel-2 multispectral bands gave rise to other covariates, and, through the *band*_*ratio* Function of the *Labgeo* package [42], in R *software* [30], all the possibilities of ratio between the bands were calculated. Filgueiras [13] called this procedure a normalized ratio between bands (NRPB) (Eq 9). Through this procedure, 45 new covariates were originated, totaling 58 independent variables.


$$NRPB=\frac{\rho_{n}-\rho_{n+1}}{\rho_{n}+\rho_{n+1}}$$ (9)

Where *ρ*_*n*_ are the surface reflectances relative to the bands of the MSI/Sentinel 2A and 2B sensor.

After the 58 covariates were obtained, predictors with high collinearity were excluded using a Pearson correlation limit of 0.98. Subsequently, the data set was filtered again to eliminate predictors that had low importance for the models using the *Recursive Feature Elimination* (RFE) function of the *Caret* package [29], in R *software* [30].

The variables under study have different spatial resolution, so it was necessary to convert all independent variables to 30 m. For this, the *resample* function of the *Raster* package [43] was applied in R *software* [30].

The data base of variables, for each pair of images, was obtained from random collection at georeferenced points. This procedure was performed using the *sampleRandom* function of the *Raster* package, present in R *software* [30]. To evaluate the performance of the models, the data set was separated into training and validation. Thus, the data sets were randomly divided into the ratio of 75% (110.442 pairs of points) for training with *k-fold* cross*-*validation (k = 10) and 25% (36.814 pairs of points) for the validation of ET_*f*_ estimate. Althoff et al. [44] state that the 75/25% ratio is a good ratio for separate training and validation sets, respectively.

### 2.5. Study of two scenarios of input of independent variables to predict ET_*f*_ considering the total area

The spectral bands of Sentinel 2A and 2B satellite and vegetation indices comprised a total of 11 variables and formed the first scenario (Table 1). In the second scenario, variables derived only from the spectral bands of Sentinel 2A and 2B satellite were combined through the NRPB. After performing the NRPB, the variables applied in the first scenario were joined, and subjected to the correlation analysis of 0.98 and the RFE Function, which made it possible to obtain 29 variables (Table 1).

**Table 1:** Combinations of input variables for each scenario.

First Scenario	Second Scenario	
ρ_2_	NRPBρ_2_ and ρ_3_ = (ρ_2_ - ρ_3_)/(ρ_2_ + ρ_3_)	ρ_2_
ρ_3_	NRPBρ_2_ and ρ_4_ = (ρ_2_ - ρ_4_)/(ρ_2_ + ρ_4_)	ρ_3_
ρ_4_	NRPBρ_2_ and ρ_5_ = (ρ_2_ - ρ_5_)/(ρ^2^ + ρ_5_)	ρ_4_
ρ_5_	NRPBρ_2_ and ρ_6_ = (ρ_2_ - ρ_6_)/(ρ_2_ + ρ_6_)	ρ_5_
ρ_6_	NRPBρ_3_ and ρ_4_ = (ρ_3_ - ρ_4_)/(ρ_3_ + ρ_4_)	ρ_6_
ρ_8A_	NRPBρ_3_ and ρ_5_ = (ρ_3_ - ρ_5_)/(ρ_3_ + ρ_5_)	ρ_7_
ρ_11_	NRPBρ_3_ and ρ_6_ = (ρ_3_ - ρ_6_)/(ρ_3_ + ρ_6_)	ρ_11_
ρ_12_	NRPBρ_4_ and ρ_8A_ = (ρ_4_-ρ_8A_)/(ρ_4_ + ρ_8A_)	ρ_12_
NDVI	NRPBρ_4_ and ρ_5_ = (ρ_4_ - ρ_5_)/(ρ_4_ + ρ_5_)	NDVI
NDWI	NRPBρ_4_ and ρ_11_ = (ρ_4_ - ρ_11_)/(ρ_4_ + ρ_11_)	EVI
EVI	NRPBρ_4_ and ρ_12_ = (ρ_4_ - ρ_12_)/(ρ_4_+ ρ_12_)	NRPBρ_8_ and ρ_6_ = (ρ_8_ - ρ_6_)/(ρ_8_ + ρ_6_)
-	NRPBρ_5_ and ρ_11_ = (ρ_5_ - ρ_11_)/(ρ_5_ + ρ_11_)	NRPBρ_8_ and ρ_7_ = (ρ_8_ - ρ_7_)/(ρ_8_ + ρ_7_)
-	NRPBρ_5_ and ρ_12_ = (ρ_5_ - ρ_12_)/(ρ_5_ + ρ_12_)	NRPBρ_8_ and ρ_8A_ = (ρ_8_ - ρ_8A_)/(ρ_8_ + ρ_8A_)
-	NRPBρ_6_ and ρ_8A_ = (ρ_6_ - ρ_8A_)/(ρ_6_ + ρ_8A_)	NRPBρ_11_ and ρ_12_ = (ρ_11_ - ρ_12_)/(ρ_11_ + ρ_12_)
**-**	NRPBρ_7_ and ρ_8A_ = (ρ_7_ - ρ_8A_)/(ρ_7_ + ρ_8A_)	-

It is important to emphasize that both scenarios are formed by the same dates and area, delimited by the pairs of coordinates X1: 660185.23; Y1: 8356189.30 (upper left corner) and X2: 693932.13; Y2: 8332533.58 (lower right corner), DATUM WGS84 reference system—UTM plane coordinate system—Zone 22S. That is, the scenarios are different only as a function of the independent variables.

The variables of the second scenario were created with the expectation that they could increase the explanatory power of the machine learning methods. These methods have good ability to find patterns in data sets with significant amount of parameters, quality and quantity of input data [3, 45, 46].

### 2.6. Performance comparison criteria

The ET_*f*_ values obtained by the models were considered as the predicted values, while the values from SAFER (ET_*f*_SAFER) were considered as observed data. Thus, the predicted values were compared with observed values using the following statistical metrics: root mean square error (RMSE, Eq 10), mean absolute error (MAE, Eq 11), systematic error (MBE, Eq 12) and coefficient of determination (r^2^, Eq 13).


$$\mathrm{RMSE}=\sqrt{\frac{\sum_{i=1}^{n}(O_{I}–P_{i}{)}^{2}}{n}}$$ (10)

$$\mathrm{MAE}=\frac{1}{n}\sum_{i=1}^{n}\left|P_{i}–O_{i}\right|$$ (11)

$$MBE=\frac{1}{n}\sum_{i=1}^{n}\left(P_{i}–O_{i}\right)$$ (12)

$$r^{2}={\left[\frac{\sum_{i=1}^{n}\left(P_{i}–P\right)(O_{i}‐\bar{O})}{\sqrt{{\left(\Sigma_{i=1}^{n}(P_{i}‐P\right)}^{2}){\left(\Sigma_{i=1}^{n}(O_{i}‐\bar{O}\right)}^{2})}}\right]}^{2}$$ (13)

Where *P*_*i*_ is the value predicted by the model; *P* is the average value predicted by the model; *O*_*i*_ is the observed value; $\bar{O}$ is the average observed value; *n* is the number of data pairs.

### 2.7. Case study

To evaluate whether Sentinel-2 is effective for obtaining the evapotranspiration fraction (ET_*f*_), the best model, in each scenario, was selected based on the statistical metrics obtained in the validation process. These models were applied to estimate ET_*f*_ in sugarcane and soybean crops irrigated by center pivots present in the delimitation of the study area on 08/06/2019 which corresponds to the 218 days of the year (DOY) and 04/02/2020 (DOY093). The land use of the center pivots was determined by using images from the *MapBiomas* product (Collection 5) [47], relative to the year 2019, available at https://mapbiomas.org.

Normalized Difference Vegetation Index (NDVI) values at different dates for cultivated areas are provided in **S1 File**. In the worksheet, the maximum, average and minimum values of NDVI of the areas vegetated with soybean and sugarcane are informed. These crops are irrigated by various central pivot equipment and the coordinates of the center of the area are informed. With these NDVI values it is possible to have an idea of the vegetative stages of agricultural crops.

In the second moment of this research, the ET_r_ calculated from the reference evapotranspiration (ET_o_) by the Penman-Monteith FAO 56 method (Eq 14) was used. This method is considered the standard for ET_o_ calculation [48, 49]. Thus, ET_r_ was used in this research to obtain the ET_r_SAFER when multiplied by the ET_*f*_SAFER to be compared with the ET_r_ estimated with the ET_*f*_ from the selected models. The empirical model of Hargreaves-Samani (HS) [50], (Eq 15) and the ET_o_Brazil product [44], were used in each approach from the multiplication of ET_*f*_Predicted, hence forming the products ET_r_HS and ET_r_ET_o_Brazil, respectively.


$${\mathrm{ET}}_{o}\mathrm{FAO}=\frac{0.408\;\Delta (R_{G}‐G)+\gamma (\frac{900}{\mathrm{Tmean}+273})U_{2}{(e}_{s}‐e_{a})}{\Delta +\gamma (1+0.34\times U_{2})}$$ (14)

$${\mathrm{ET}}_{o}HS=\propto (T_{\max}–T_{\min}{)}^{\beta }\;(T_{\mathrm{mean}}+17.8)R_{o}\times 0.408$$ (15)

Where *ET*_*o*_ is reference evapotranspiration (mm d^-1^); *Δ* slope of the vapor pressure curve versus temperature (kPa °C^-1^); *R*_*G*_ net radiation at the crop surface (MJ m^-2^ d^-1^); *G* is the soil heat flux density (MJ m^-2^ d^-1^); *γ* is the psychrometric constant (0.0677 kPa °C^-1^); *T*_*max*_ is the maximum air temperature (°C^-1^); *T*_*min*_ is the minimum air temperature (°C^-1^); *T*_*mean*_ is the mean air temperature (°C^-1^); *U*_*2*_ is the average wind speed at 2-m height (m s^-1^); *e*_*s*_ is the vapor saturation pressure (kPa); *e*_*a*_ is the actual vapor pressure (kPa); ∝ is an empirical parameter (its original value of 0.0023 was used); *β* is an exponential empirical parameter (its original value of 0.5 was used); *R*_*o*_ is extraterrestrial solar radiation (MJ m^-2^ d^-1^).

ET_o_ by the Penman-Monteith FAO 56 method was calculated from the *BrazilMet* package [51], in R statistical *software* [30]. The *BrazilMet* package used daily data from the automatic station of the National Institute of Meteorology (INMET) [52], installed in the municipality of Itapaci-GO (A015), located 20.5 km away from the center of the study area. The coordinates of station A015 are X:656990.30; Y:8343368.99, DATUM WGS84 reference system—UTM plane coordinate system—Zone 22S. ET_o_HS was calculated using data from the aforementioned station, with the exception of extraterrestrial radiation, which was spatially calculated in R statistical software (Eq 16).


$$R_{o}=\frac{24\times (60)}{\pi }\times S_{c}\times D_{r}\times (\hat{H}\times \mathrm{sen}\emptyset \times \mathrm{sen}\delta +\cos\emptyset \times \cos\delta \times \mathrm{sen}\hat{H})$$ (16)

where: *R*_*o*_ is extraterrestrial solar radiation (MJ m^-2^ d^-1^); *S*_*c*_ is the solar constant that is equivalent to 0.0820 (MJ m^-2^ min^-1^); *D*_*r*_ is the relative distance between the earth and the sun in astronomical units; *Ĥ* is the hour angle at sunrise; ∅ is latitude; *δ* is solar declination.

According to Iqbal [53], the relative distance between the earth and the sun in astronomical units can be calculated by Eq 17.


$$D_{r}=1+0.033\cos\left(J\times \frac{2\times \pi }{365}\right)$$ (17)

where: *J* is the day in the Julian calendar.

The ET_o_Brazil product covers Brazil as a whole and provides daily ET_o_ information that was estimated spatially using data from 849 weather stations [44]. This product has a spatial resolution of approximately 10 km. It is worth pointing out that, for an effective application of the ET_o_Brazil product, it was necessary to use the *disaggregate* function of the *Raster* package [43], present in R software [30].

## 3. Results and discussion

### 3.1 Validation of models for evapotranspiration fraction (ET_*f*_) estimation

**Fig 3:**
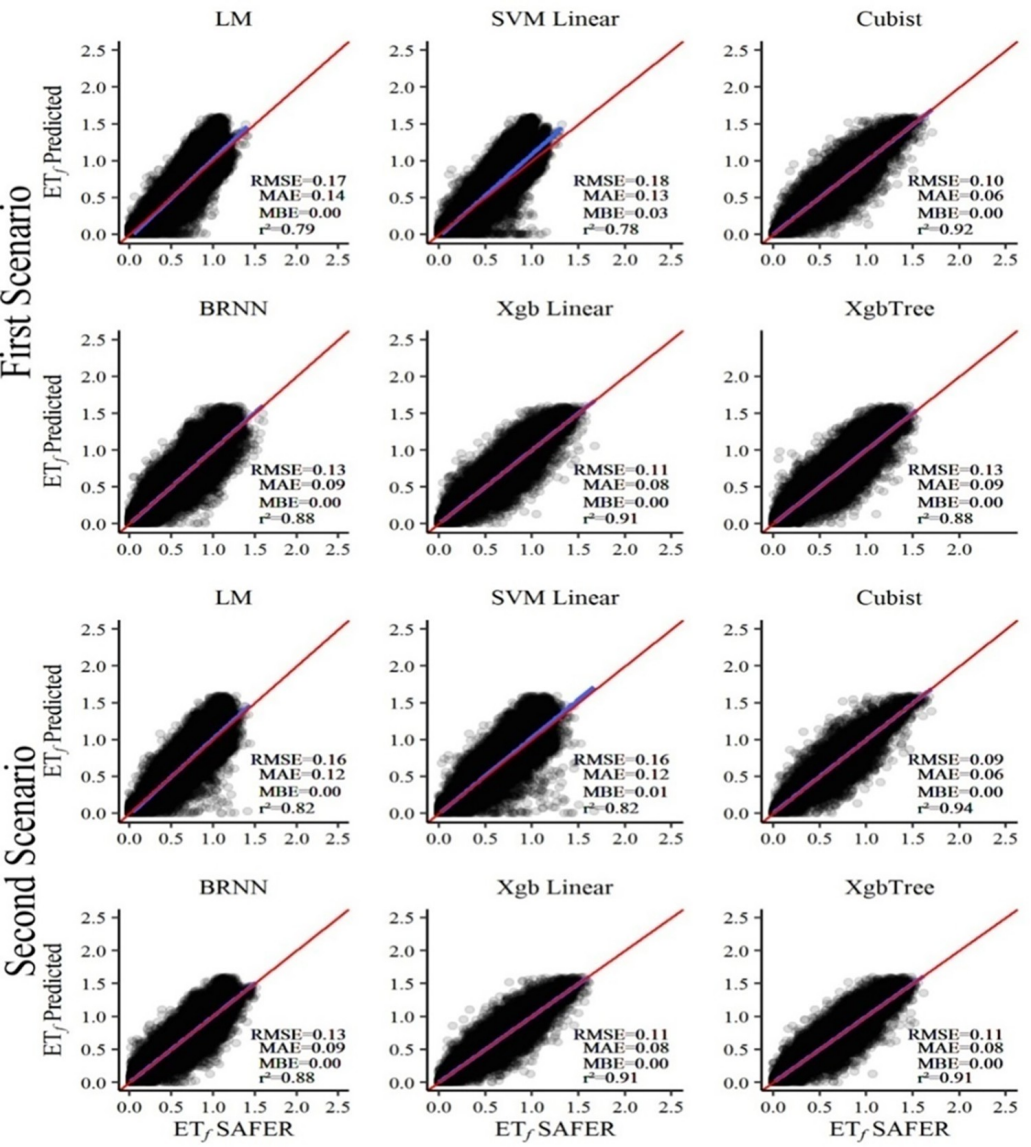
Daily values of evapotranspiration fraction (ET_*f*_) predicted by different approaches (scenarios 1 and 2) compared with values obtained by SAFER from Landsat-8 images in the period from 2016 to 2018.

To evaluate the possible improvement in the performance of prediction models, the approach of two different scenarios was chosen. The scenarios refer to different combinations of input variables to estimate ET_*f*_ through regression algorithms, since the intention is to fit a simple and accurate model. This made it possible to know, in each scenario, the regression algorithm that had the highest capacity to predict ET_*f*_. The performance of the methods for predicting ET_*f*_ for the different scenarios is shown in Fig 3.

For the authors Chandel et al. [54] and Zhang and Zhou [55] the vegetation indices that have a band with a short infrared or red wavelength show greater sensitivity to the water content in the leaves. The presence of the indices generated through the NRPB that were selected in the second scenario, which have a band in the short or red infrared, are seven. This may be one of the arguments that affirms the superior predictive capacity of the second scenario, not ruling out that the greater number of variables influenced the result.

By analyzing Fig 3, it can be noted that both scenarios promoted good performances, because the models showed accuracy (low values of MAE and RMSE) and small presence of outliers due to the proximity of RMSE and MAE. The SVM Linear model overestimated ET_*f*_ and the other algorithms had MBE values equal to zero, thus showing that there was no overestimation. Therefore, in general, all models showed good accuracy (high values of r^2^). It can also be observed in each method that the line of the fitted linear model is similar to the ideal line (1:1).

The values obtained from the statistical indicators show that the predicted values (ET_*f*_Predicted) are positively correlated with the data derived from ET_*f*_SAFER, that is, they had the same linear trend in both scenarios. Although the first scenario contains fewer input variables in the models, the statistical results obtained indicate high predictability of the methods under these conditions.

It is also noticed that the models of the second scenario differed slightly from those of the first scenario, showing a reduction in RMSE values and positive increments in the values of r^2^, except for the BRNN and XgbLinear models, which had the same metrics in both circumstances. However, the cloud of points is different, being more concentrated around the 1:1 line in the second scenario. It is worth mentioning that, when the models of the second scenario were run, the time required for their training increased more than 70 times in comparison to the model with lowest machine cost, but it did not lead to major changes in the performance of the methods. Thus, computational time, which is highly dependent on the speed of the computer processor, should be considered as an important element of comparison to process large amounts of data in a short time [45, 46].

The MLR and SVM Linear methods showed similar statistical behaviors in the performance of ET_*f*_ prediction in both scenarios and have lower explanation capacity when compared with the others. However, the SVM Linear method had the highest machine cost, taking up to 70 times longer to be trained, when compared to the MLR, which used the shortest computational time among all algorithms. Cubist, which has better statistical results, had a training time approximately 20 times longer than the time spent by the MLR. It is worth mentioning that in the present study a conventional computer was used to perform the training. Perhaps, if it was necessary to reduce the time of the procedures, it could use the cloud processing to adapt the time to the demand.

Given the analyses that the two scenarios obtained good results, it is necessary to verify the performance of the models that best fitted the ET_*f*_SAFER data on each occasion. Thus, the two best models in descending order are the Cubist and the XgbLinear, with slight superiority for the Cubist. XgbTree is the third best model in the second scenario, while in the first scenario it also promoted good performance, with statistical indices equal to those of BRNN. As the performance of the models is considered satisfactory in both strategies, the subsequent analyses and discussion were based on the best model of each scenario.

The selection of an algorithm with excellent accuracy is essential for the obtaining of estimates and later practical application for the prediction of ET_r_, which is an important parameter for irrigation management. Despite the similarity in the performance of the two best models, the Cubist was chosen because it had a slight advantage in all scenarios studied. Also in this context, the variations of RMSE and r^2^ values in the validated modeling of Cubist in the first and second scenarios were from 0.10 to 0.09 (-10%) and from 0.92 to 0.94 (2.13%), respectively. In addition, the magnitude of RMSE relative to the mean of ET_*f*_SAFER was equivalent to 18.86 and 16.67% for the first and second scenarios, respectively.

The ET_*f*_Predicted by the Cubist method, in the present study, is in accordance with the results obtained by Filgueiras et al. [51], who conducted a study with six regression algorithms to estimate ET_r_ without thermal information, using 40% (NDVI and SR) of the independent variables from spectral information of the MODIS sensor on board the TERRA platform. The authors found RMSE and r^2^ with the help of the Cubist model of 0.40 and 0.92, respectively, which shows the robustness of the tool in ET_r_ estimation. Thus, these authors indicated it as the best algorithm to predict ET_r_ in that situation.

The Cubist model is based on the modified regression trees theory and its principle is to generate prediction models from rule-based systems. These rules are created using training data and each has a multivariate linear model, which are overlapped and arranged in ascending order of predicted mean values [56]. Cubist has been widely applied in academic studies in a variety of areas from satellite images. In this context, several authors conclude that this method provides great robustness and greater accuracy when estimating ET when compared to other traditional machine learning algorithms, such as multiple linear regression, principal component analysis, artificial neural networks, support vector machine, among others [44, 53, 57–59].

When using the methodology applied in this study, the estimation of ET_*f*_ through the MSI sensor becomes feasible and these results can be used in temporal studies containing information derived from Landsat-8, since the model was based on the information of this product. This fact enables the presence of a higher frequency of information and, therefore, it is possible to monitor the evapotranspiration of a crop with a substantially lower image periodicity, as mentioned by Filgueiras et al. [13].

### 3.2. Estimation of evapotranspiration fraction (ET_*f*_) in areas irrigated by center pivot

**Fig 4:**
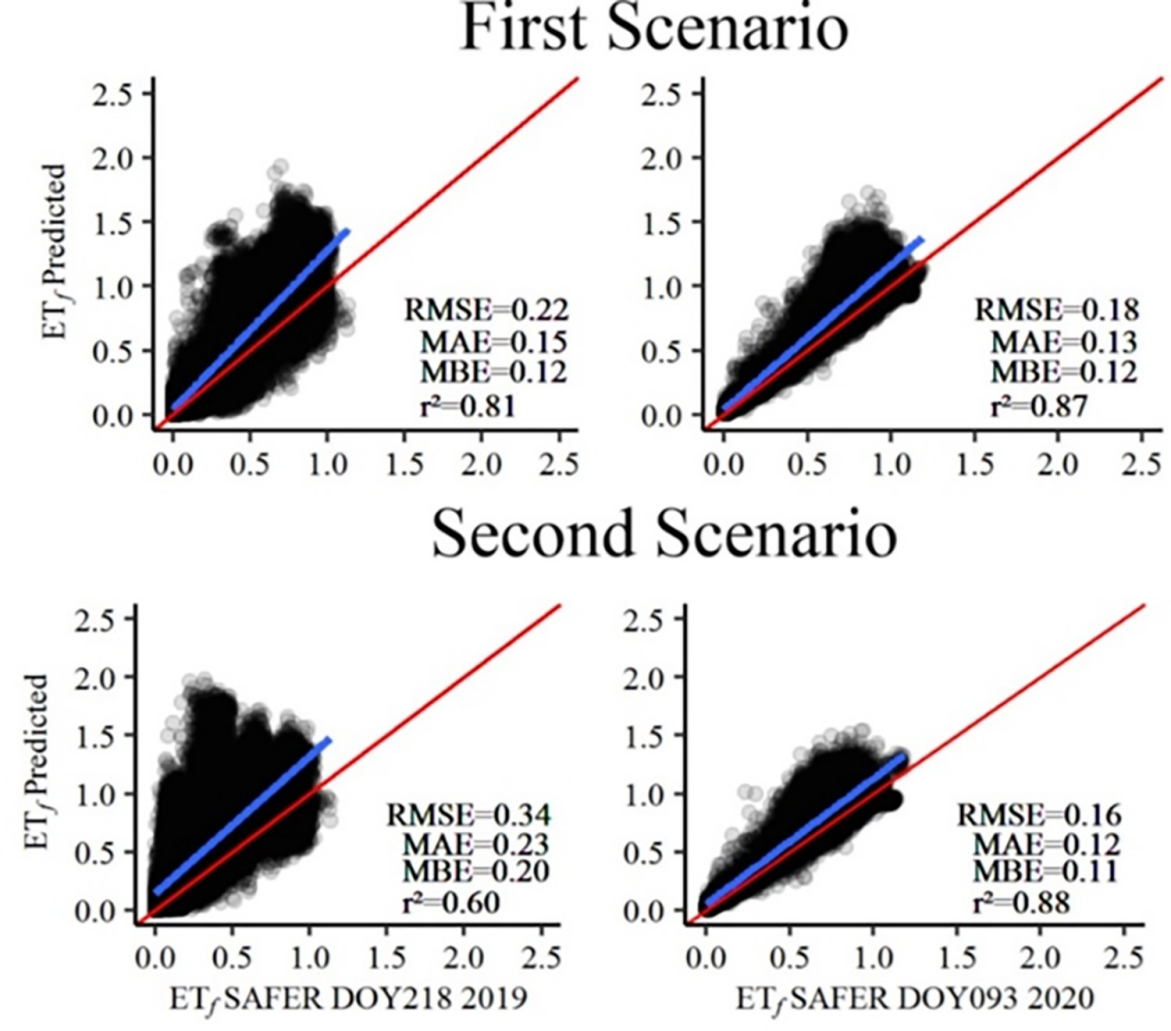
Application of the Cubist model for prediction of evapotranspiration fraction (ET_*f*_) in areas with center pivots compared with values obtained by SAFER with Landsat-8 images for the days 08/06/2019 (DOY218) and 04/02/2020 (DOY093).

As the objective of the proposed ET_*f*_ modeling was to apply the trained and validated model to estimate ET_r_ in agricultural areas a posteriori, it is convenient to analyze the scatter plots of the applicability of the model only in areas irrigated by center pivots. This analysis is important to know the sensitivity of the values of ET_*f*_Predicted versus ET_*f*_SAFER, and to know the generalization capacity of the models in predictions for dates not used in the training (Fig 4)—which simulates the applicability of the model in practice. When analyzing the MBE, it can be observed that the Cubist model, in this application, overestimated the values in both scenarios and on the two days 08/06/2019 (DOY218) and 04/02/2020 (DOY093), with values ranging from 0.11 to 0.20. For Stone [60], the closer the MBE value is to zero, the better the performance of the model under study.

The data on the date corresponding to DOY218 showed poorer behavior compared to that of DOY093 in both scenarios. This is highly associated with land use on this day, showing low mean value of NDVI, ranging from 0.1 to 0.8, with an average of 0.4. For DOY093, NDVI values are within the range from 0.1 to 0.9, with an average of 0.7. NDVI is directly related to the photosynthetic components of the plant, and the presence of low NDVI values indicates that plants, in most center pivots, had a small leaf area or are at the physiological maturity stage [40, 61].

It is important to highlight that NDVI is one of the variables used for the calculation of ET_*f*_SAFER. In this scenario, low values of NDVI result in values closer to zero for ET_*f*_SAFER, and this occurred around 45% of the area under study for the day referring to DOY218, when ET_*f*_SAFER ranged from 0 to 0.40. ET_*f*_Predicted, on DOY218, showed values closer to one in both scenarios. Therefore, the errors were relatively higher and the accuracy was lower when compared to the image referring to DOY093, which had ET_*f*_SAFER values from 0.45 to 0.90 in more than 70% of the area.

Corroborating Carpintero et al. [62], the results found in the application of the model in areas irrigated by center pivots are possibly linked to the oscillations that occur in the dynamics of land use in agricultural areas, which ends up causing different spectral responses in the data captured by the sensors. Moreover, the relationships observed under different conditions of the study area may not be able to properly generalize, with the same performance, since the behavior of regression algorithms may be highly specific to the site [63, 64].

Dou and Yang [46] also evaluated the performance of models developed to estimate evapotranspiration in four different terrestrial ecosystems (forest, pasture, cropland and wetland). The authors verified that the predictive capacity of the four machine learning algorithms varied with the different ecosystems. These results are justified by the fact that evapotranspiration is influenced by land use and cover [65].

### 3.3. Estimation of actual evapotranspiration (ET_r_) using two approaches in ET_o_ calculation in two agricultural crops

**Fig 5:**
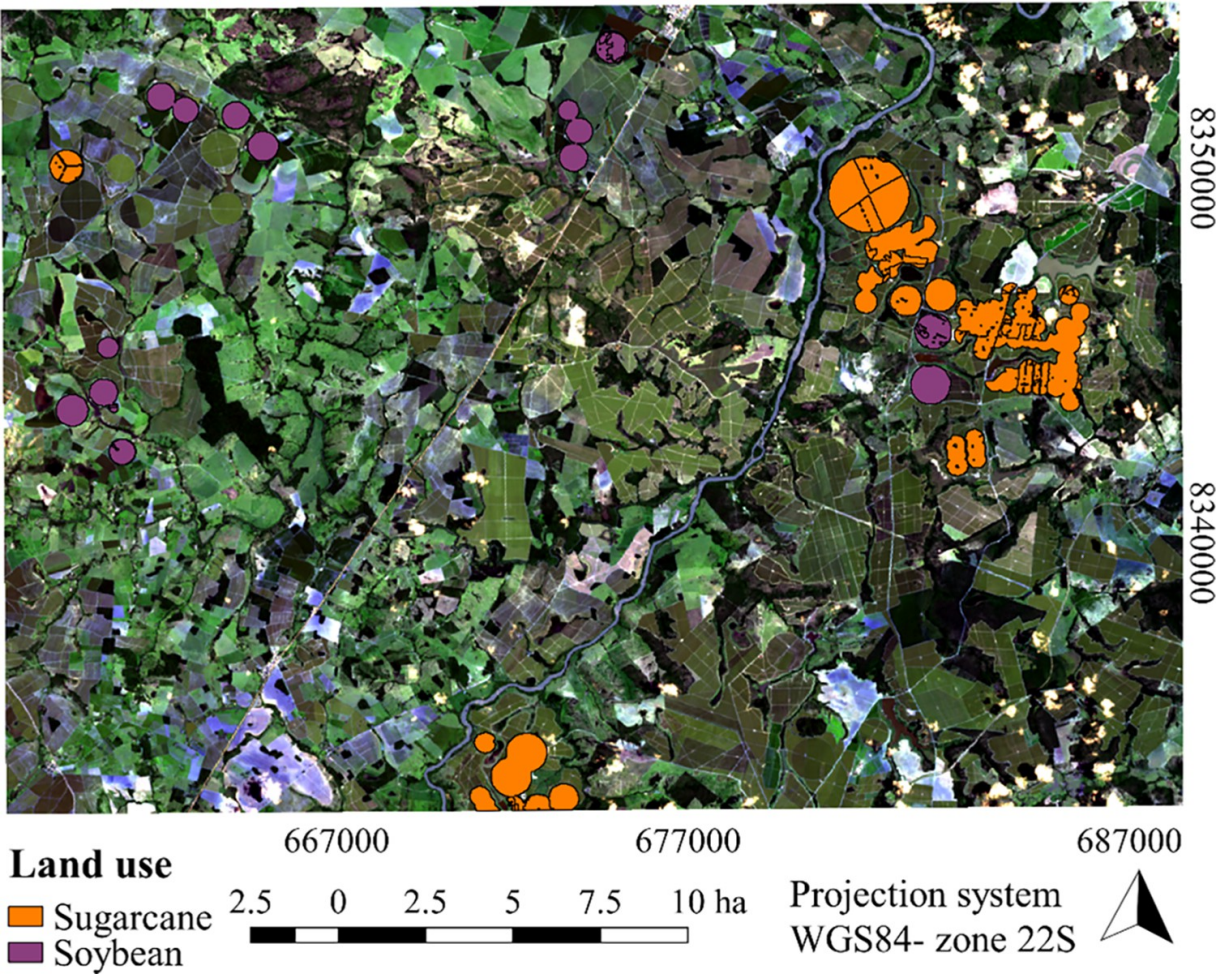
Land use and land cover classification from *MapBiomas*, collection 5. The figure was elaborated using the open source software QGIS [25]—using an RGB image composite generated from the Landsat 8 satellite images that were downloaded from https://earthexplorer.usgs.gov/. *Landsat-8 image courtesy of the U*.*S*. *Geological Survey* according to [26].

**Fig 6:**
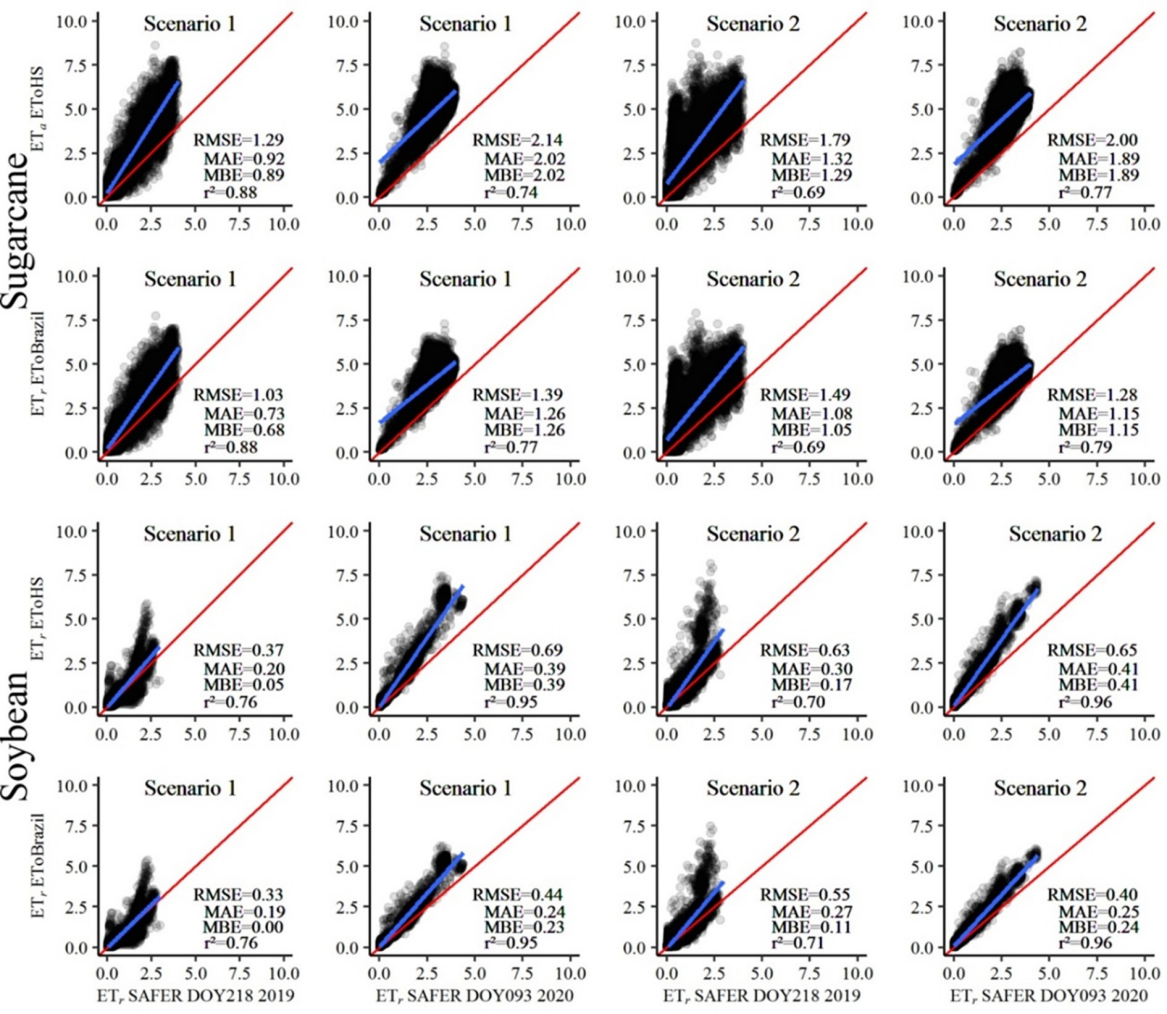
Application of the Cubist model for the prediction of actual evapotranspiration (ET_r_) in areas with center pivots using sugarcane and soybean crop, obtained through two approaches, (ET_r_ET_o_HS and ET_r_ET_o_Brazil), and compared with values obtained by (ET_r_SAFER) for the days 08/06/2019 (DOY218) and 04/02/2020 (DOY093).

Evapotranspiration is an important factor for irrigation management, while land use and cover acts directly on this estimate. Thus, the land use and cover data for the center pivots, from *MapBiomas*, were accessed (Fig 5). It was verified that 85% of the center pivots were occupied with sugarcane, while the rest were occupied with soybean. From this information, ET_r_ was calculated for each crop and compared using two approaches: ET_r_SAFER vs ET_r_ET_o_HS and ET_r_SAFER vs ET_r_ET_o_Brazil for the center pivots on the days DOY218 and DOY093 in both scenarios (Fig 6).

The study with both approaches makes it possible to know the best method of ET_o_ to calculate ET_r_ for the conditions and methodologies applied in the present study, which can make it possible to monitor the crop with a substantially higher image frequency, as well as making the observation of the spatial-temporal dynamics of evapotranspiration practical. When comparing the mean difference of the statistical metrics of the second strategy with those of the first one on the two days under study, in the approaches for any land use, it is verified that the first scenario has greater precision in ET_r_ estimation, with some exceptions. The exceptions were for the comparisons ET_r_SAFER vs ET_r_ET_o_Brazil for sugarcane and ET_r_SAFER vs ET_r_ET_o_HS and ET_r_SAFER vs ET_r_ET_o_Brazil on DOY093 in areas with soybean crop.

In this context, it is observed that the estimated values of ET_r_ for sugarcane have higher errors and lower r^2^ when compared with those for soybean. This occurred mainly for ET_r_SAFER vs ET_r_ET_o_HS, whereas, if compared with ET_r_SAFER vs ET_r_ET_o_Brazil, the error indicators are relatively better in both crops. However, sugarcane still has lower metrics. This behavior may be explained by the fact that this agricultural crop does not have the same window dynamics as that of soybean, which is more rigid. This is because sugarcane is a semi-perennial crop, with a production cycle of six to seven years.

From the spectral reflectances in the areas irrigated by the center pivots, it is observed that there is a more heterogeneous behavior of sugarcane plants and this is reflected in the prediction of ET_r_, since the independent variables directly influence ET_*f*_. This makes it possible to highlight that the plants have greater spectral variation, when analyzing the center pivots with this crop, different from what is verified with the center pivots with soybean crop (in which the spectral behavior is more homogeneous). It is worth pointing out that the dominant factors that control leaf reflectance are pigments, cell structure and water content [66–68]. These factors depend on the crop, physiological stage and management of the cultivation area.

Also in Fig 5, all approaches and land use in the areas irrigated by the center pivots showed an overestimation, except for the comparison ET_r_SAFER vs ET_r_ET_o_Brazil for soybean in the first scenario, on the day corresponding to DOY218. In this period, the areas cultivated with sugarcane also stood out, with higher values of MBE (0.68 to 2.02 mm d^-1^), while for soybeans, the values are between 0.00 and 0.41 mm d^-1^. Santos et al. [4], when working with the SAFER algorithm and testing various ET_o_ methods to predict ET_r_, found that MBE is directly related to the values estimated using ET_o_.

In this study, when analyzing the scatter plots for sugarcane, it was verified that, regardless of the approach used to estimate ET_r_, it is pointing to higher water demand compared with ET_r_SAFER, especially ET_r_ET_o_HS, which would have a direct impact on irrigation management. This indicates that obtaining ET_o_ the HS method is not an adequate strategy to map ET_r_ for sugarcane under the studied conditions, so this is not a recommended methodology.

However, if the producer chooses to apply the average water depth, the ideal is to use the ET_r_ET_o_Brazil approach. Nonetheless, some areas will be over- or under-irrigated, which will impact yield or result in waste of water resources and may even lead to an increased incidence of agricultural diseases [1, 4].

Santos et al. [4] estimated the ET_r_ of sugarcane crop using SAFER and the ET_o_ data calculated by the HS model. Thereby, verified an overestimation with this strategy, suggesting greater water demand compared with ET_r_SAFER, which was estimated by ET_o_ calculated by Penman-Monteith FAO56. Therefore, the methodology that uses the HS model was not recommended by the authors. Other authors have also studied the HS method and verified overestimates of the ET_o_ values, indicating improvements in the efficiency of the method only after calibration of the model [69, 70].

As observed in Fig 6, the different ET_o_ estimation methodologies directly impacted the prediction of ET_r_ in both scenarios. Therefore, it is clear that the Cubist model, for the first scenario, and the approach ET_r_ET_o_Brazil respond better when one is working with soybean crops irrigated by center pivots. This approach has real potential to improve the ET_r_ estimation in time and space and makes it possible to monitor water demand more frequently during the crop cycle.

## 4. Conclusions and recommendation

The study explored six regression algorithms (MLR, SVM Linear, Cubist, BRNN, XgbLinear and XgbTree) in two different data input scenarios. The first scenario was worked with 11 independent variables, while the second scenario, with 29. Regardless of these scenarios, the Cubist model should be recommended for predicting the evapotranspiration fraction, since it showed the best performance metrics, as well as the greater computational cost compensation required for validation.

The methodology applied in this study made it possible to analyze the actual evapotranspiration (ET_r_) using the ET_r_ET_o_HS and ET_r_ET_o_Brazil approaches in the areas under center pivots with sugarcane and soybean crops. The first scenario and the ET_o_Brazil product comprise the recommended strategy to estimate ET_r_ for both agricultural crops, but the best performances were obtained in soybean crops. In contrast, the Hargreaves-Samani equation overestimated ET_r_ and led to greater errors, especially in irrigated sugarcane crops.

The findings found in this study are promising and of great importance for water management in irrigation, especially in center pivots with soybean crop. Thus, this study proved the potential of using machine learning approaches, considering their effectiveness and ease of implementation. However, it must be emphasized that this research is unprecedented and should be tested in other areas to verify whether the behaviors of the two agricultural crops are similar to the results found here.

Based on the findings, the subsequent recommendations are forwarded:

- More studies should be carried out to confirm our results and reduce errors in the ET_r_ estimation. In this sense, the introduction of meteorological data from satellites can be used in future studies. These data with daily temporal resolution can provide a better explanation of the phenomenon we are monitoring.
- The authors also suggest further studies to assess whether the proposed methodology is good enough for ET_r_ spatialization for precision agriculture. This is a point that was not addressed in the present research and that would require evaluations to understand whether the methodology could also be applied for this purpose.

## Supporting information

S1 File Maximum, average and minimum values of Normalized Difference Vegetation Index (NDVI) in areas cultivated with soybean and sugarcane and irrigated by center pivot at different dates. (XLSX)
